# Dampening the DAMPs: How Plants Maintain the Homeostasis of Cell Wall Molecular Patterns and Avoid Hyper-Immunity

**DOI:** 10.3389/fpls.2020.613259

**Published:** 2020-12-17

**Authors:** Daniela Pontiggia, Manuel Benedetti, Sara Costantini, Giulia De Lorenzo, Felice Cervone

**Affiliations:** ^1^Dipartimento di Biologia e Biotecnologie “Charles Darwin,” Sapienza Università di Roma, Rome, Italy; ^2^Dipartimento di Medicina Clinica, Sanità Pubblica e Scienze della Vita e dell’Ambiente, Università degli Studi dell’Aquila, L’Aquila, Italy

**Keywords:** growth-defense trade-off, Berberine bridge enzyme-like (BBE-like) proteins, cell wall DAMPs, oligo-saccharide oxidase, oxidized oligogalacturonides, oxidized cellodextrins

## Abstract

Several oligosaccharide fragments derived from plant cell walls activate plant immunity and behave as typical damage-associated molecular patterns (DAMPs). Some of them also behave as negative regulators of growth and development, and due to their antithetic effect on immunity and growth, their concentrations, activity, time of formation, and localization is critical for the so-called “growth-defense trade-off.” Moreover, like in animals, over accumulation of DAMPs in plants provokes deleterious physiological effects and may cause hyper-immunity if the cellular mechanisms controlling their homeostasis fail. Recently, a mechanism has been discovered that controls the activity of two well-known plant DAMPs, oligogalacturonides (OGs), released upon hydrolysis of homogalacturonan (HG), and cellodextrins (CDs), products of cellulose breakdown. The potential homeostatic mechanism involves specific oxidases belonging to the family of berberine bridge enzyme-like (BBE-like) proteins. Oxidation of OGs and CDs not only inactivates their DAMP activity, but also makes them a significantly less desirable food source for microbial pathogens. The evidence that oxidation and inactivation of OGs and CDs may be a general strategy of plants for controlling the homeostasis of DAMPs is discussed. The possibility exists of discovering additional oxidative and/or inactivating enzymes targeting other DAMP molecules both in the plant and in animal kingdoms.

## Introduction

The cell wall represents the interface between plants and the environment and acts as a physical barrier to protect plants against biotic and abiotic stresses. Main components of the cell wall are the polysaccharides cellulose, hemicellulose, and pectin, which interact among themselves and with lignin to form a complex structure that influences the shape, rigidity, growth, and differentiation of plant cells (Bacete and Hamann, 2020; Zhang and Zhang, 2020). All three types of polysaccharides are also a repository of extracellular damage-associated molecular patterns (DAMPs), potentially released during microbial infections or upon mechanical damage (De Lorenzo et al., 2018; Hou et al., 2019). DAMPs are likely to be released also upon cell wall remodeling that occurs during plant growth and development. Microlesions caused, for example, by cell expansion, lateral root formation, or organ abscission, may cause cell wall damage and affect the so-called cell wall integrity (CWI; Anderson and Kieber, 2020; Gigli-Bisceglia et al., 2020). In these cases, endogenous cell wall degrading enzymes (CWDEs) can release, at low levels, oligosaccharide fragments of the same nature as those accumulated in larger quantities during more destructive mechanical injuries or plant cell wall degradation caused by pathogen-encoded CWDEs (De Lorenzo et al., 2018, 2019). Some DAMPs are recognized by pattern-recognition receptors and are capable of inducing pattern-triggered immunity (PTI) even in the absence of infection (De Lorenzo et al., 2018).

Unlike mammals, plants do not have an adaptive immune system but, instead, entirely rely on the ability of individual cells to recognize pathogens and to activate defense responses (Gust et al., 2017). The plant cell must be able to discriminate between the possibilities that cell wall degradation is caused by a physiological or a pathological accumulation of DAMPs. The time of formation, the appropriate concentration, and the correct distribution of the active molecules may help distinguish a pathological from a physiological event. In the former case, plants need to respond quickly, intensively, and systemically to the danger, whereas in the latter case, they may only need to activate local immune responses, preventing a massive immune response that may hamper growth (Li et al., 2020). In general, plants cannot dissipate too much energy in defense *vs*. growth and need to maintain the correct growth-defense trade-off. To this purpose, they require continuous balancing of the defense pathways and signaling.

**Figure 1:**
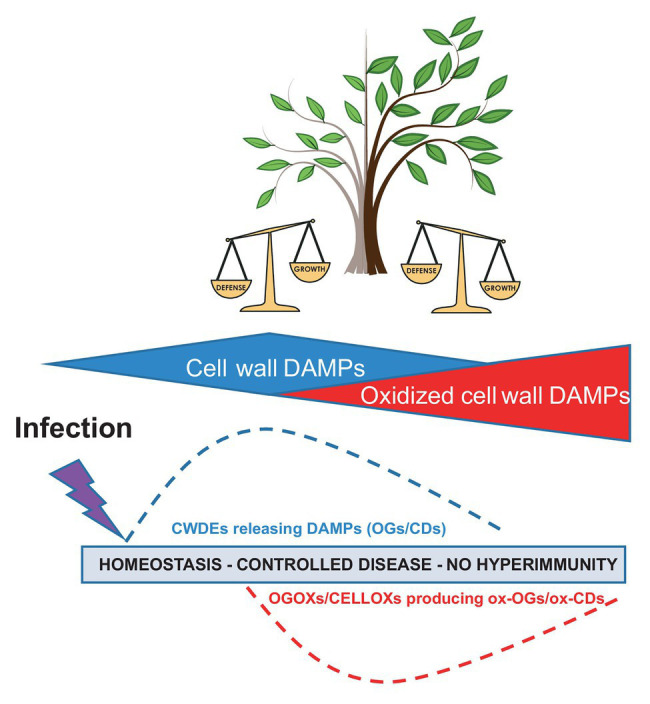
Damage-associated molecular patterns (DAMPs) and oxidized DAMPs in immunity, homeostasis of signals, and “growth-defense trade-off” (modified from Relja and Land, 2020). CWDEs, cell wall degrading enzymes; OGs, oligogalacturonides; CDs, cellulose fragments; ox-OGs, oxidized oligogalacturonides; and ox-CDs, oxidized cellulose fragments.

Hyper-immunity generated by DAMPs can cause deleterious effects in plants like in animals. Uncontrolled or prolonged production of DAMPs may promote hyperinflammation or chronic inflammation through the adaptive immune system in animals (Land, 2020), whereas in plants, it mainly causes hypersensitivity and death of individual cells and reduced growth of the entire organism. In this review, we discuss how several extracellular DAMPs released from the polysaccharides of the plant cell wall may be kept under homeostatic control by oxidizing/inactivating enzymes (Figure 1) and how a similar mechanism may have general relevance for other DAMPs.

## Dynamics of the Cell Wall and Release of Regulatory Fragments

In order to provide structural support during growth and protection against diseases, the composition and structure of the plant cell wall is continuously changing during development or upon biotic and abiotic stresses (Vaahtera et al., 2019). During both development and disease, CWI is often compromised and cell wall components undergo dynamic changes (Gigli-Bisceglia et al., 2020). These changes may cause a direct enzymatic release of fragments possessing growth-regulating/DAMP activity from each of the major wall components. The impairment of CWI itself could function as a mechano-sensory signal of damage that may also regulate cellular events, such as meristem patterning and the cell cycle (Bacete and Hamann, 2020). Notably, microtubule organization, orientation, and deposition of cellulose microfibrils are reorganized, and several CWDEs are activated in response to mechanical stimuli (Vaahtera et al., 2019).

Pectin, which is abundant in the middle lamella and in the primary walls, influences wall porosity and thickness and may play a role in the maintenance of CWI due to its marked sensitivity to mechanical deformation. A recent model indicates that cell shape and expansion as well as the turgor force exerted by the cell wall are driven by 15–30 nm nanofilaments of homogalacturonan (HG), the main component of pectin (Haas et al., 2020). These nanofilaments, located perpendicularly to the cotyledon surface, expand upon demethylation (Haas et al., 2020). Among the cell wall components, pectin appears to be more readily degraded upon tissue damage and microlesions that may arise during growth and development (Gigli-Bisceglia et al., 2020). Pectin also represents the first target of CWDEs produced by invading pathogens. The breakdown fragments of HG, the oligogalacturonides (OGs), act as regulators of plant growth and development and, if accumulated at high concentration, act as elicitors of plant defense (DAMPs). Interestingly, an alteration of the lignin content of cell walls has been reported to induce the expression of genes encoding CWDEs, such as pectate lyase, xyloglucan (XG) endo-transglycosylases, and ARABIDOPSIS DEHISCENCE ZONE POLYGALACTURONASE (ADPG1). Especially, ADPG1 may be responsible for the release of elicitor-active OGs, which could explain why a reduction in lignin content often enhances disease resistance rather than reducing it (Gallego-Giraldo et al., 2020).

Xyloglucans, hemicelluloses interacting with cellulose through hydrogen bonds, may control the rate of cell wall expansion and plant growth (York et al., 1985; Cosgrove, 2018). XG-derived fucose-containing oligosaccharides have been reported to display growth regulating activity (York et al., 1985; Zablackis et al., 1995). On the other hand, XG-derived fragments have been reported to act as DAMPs (Claverie et al., 2018).

Arabinoxylan (AX), another hemicellulose present in both the primary and secondary walls, consists of copolymers of two pentoses: arabinose and xylose. Very recently, by analyzing mutants in Arabidopsis Response Regulators (ARRs), which mediate cytokinin signaling and modulate the disease resistance (Bacete and Hamann, 2020), it was demonstrated that the AX-derived pentasaccharide 3^3^-*α*-L-arabinofuranosyl-xylotetraose (XA3XX) triggers a strong immune response in Arabidopsis and enhances disease resistance of some crop plants (Mélida et al., 2020).

Cellulose crystalline microfibrils are present at high levels together with hemicelluloses and lignin in the secondary wall (Cosgrove, 2014). The inhibition of cellulose biosynthesis triggers NADPH oxidase-dependent production of hydrogen peroxide and lignin in the elongation zone of the root. Lignin deposition is a compensatory response to reinforce cell walls that occur upon cell wall modifications or degradation (Denness et al., 2011; Vaahtera et al., 2019). Arabidopsis mutants impaired in the perception of cell wall damage and reactive oxygen species (ROS) perception/signaling show decreased or absent lignin deposition in response to cell wall damage, suggesting that the damage causes the release of DAMPs such as OGs that activate lignification to reinforce the secondary wall (Gallego-Giraldo et al., 2018, 2020). Most of the genes involved in cell wall damage are also implicated in responses to biotic stresses (Denness et al., 2011). Cellulose breakdown products, i.e., cellodextrins (CDs), also act as elicitors of plant defense (Aziz et al., 2007; Souza et al., 2017; Johnson et al., 2018).

Recently, a non-branched 1,3-*β*-D-(Glc)-hexasaccharide has been identified as a major fungal microbe-associated molecular pattern (MAMP). Because linear β-1,3-glucans are present in plants and accumulate as callose papillae at the site of infection (Albersheim et al., 2011; Chowdhury et al., 2014), the possibility exists that this type of oligosaccharide is released during callose synthesis or degradation to act as a DAMP (Mélida et al., 2018).

Mannans are mainly considered as storage polysaccharides that provide energy for the growing seeds, and their presence and functional importance as structural components of both primary and secondary walls has been highlighted by several authors (Schröder et al., 2009; Marcus et al., 2010). Recently, mannan oligosaccharides (MOS) have been reported as novel cell wall DAMPs (Zang et al., 2019).

**Figure 2:**
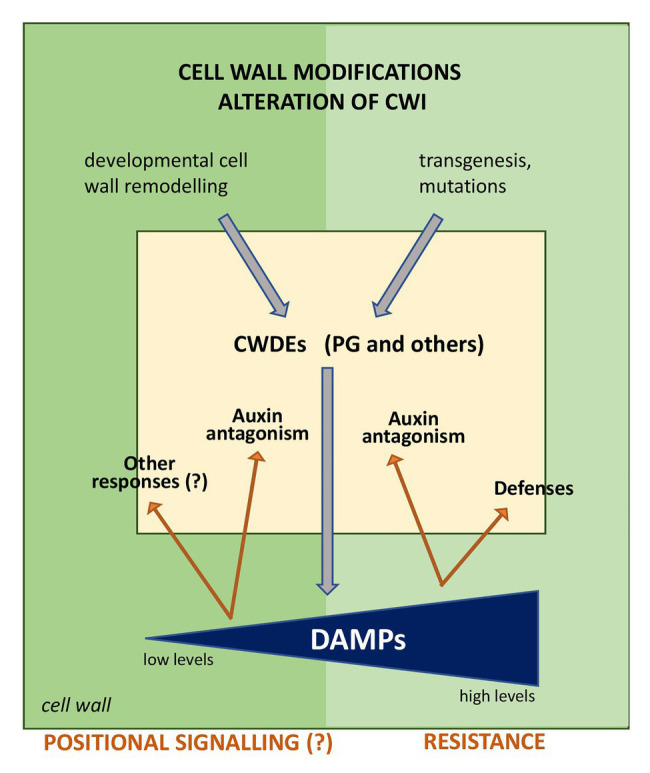
Possible outcomes of the release of DAMPs during alteration of cell wall integrity (CWI). Alterations of the cell wall composition due to transgenesis (for example, reduced lignin content) or mutations lead to a signaling cascade that induces the expression of endogenous cell wall degrading enzymes (CWDEs), among which a polygalacturonase (PG) may be responsible for the release of oligogalacturonides (OGs) that activate defense responses. In normal conditions, i.e., in the absence of pathogens, alterations of CWI during growth, and developmental events, such as cell expansion and division, wall remodeling, secondary root formation, abscission, etc. may be mediated by and/or induce the expression of CWDEs, including PGs. CWDEs release wall fragments that may act as signals for the regulation of growth in a short range cell-cell communication (positional signaling). The question mark indicates events to be elucidated.

In conclusion, the plant cell wall is a dynamic structure that during growth and development can depolymerize its components by auto-degradation, recycle the released fragments, and rebuild the entire structure (Barnes and Anderson, 2018). Alterations of cellulose, xylan, glucuronoxylan, pectin, xyloglucans, and lignin often enhance disease resistance due to their recalcitrance to pathogen-mediated degradation, or, as reported in the case of altered lignin content, provoke the release of DAMPs that activate defense responses (Gallego-Giraldo et al., 2020). During growth, in the absence of pathogens, released fragments, like OGs, may play a physiological role as growth regulators and be recruited as DAMPs if a pathological event occurs (Figure 2).

## The Mode of Action of Cell Wall Damps

### Oligogalacturonides

The best described wall-associated DAMPs are the OGs, which derive from the partial degradation of HG (Cervone et al., 1989; Orozco-Cardenas and Ryan, 1999) and likely act as indicators of CWI under stress conditions (Rojo et al., 1999; Aziz et al., 2004; De Lorenzo et al., 2011; Ferrari et al., 2013; Savatin et al., 2014). OGs antagonize auxin (Branca et al., 1988; Bellincampi et al., 1995) by inhibiting the transcription of auxin-induced genes belonging to the IAA and SAUR families as well as the activity of the auxin-responsive promoter DR5 (Savatin et al., 2011). During microbial infections, the accumulation of OGs is facilitated by the interaction of microbial polygalacturonases (PGs) with specific PG-inhibiting proteins (PGIPs; Kalunke et al., 2015; Haeger et al., 2020). Notably, the transgenic expression of a PGIP-PG chimera referred to as the “OG-machine” is able to produce *in vivo* endogenous OGs, enhancing Arabidopsis resistance against *Botrytis cinerea*, *Pectobacterium carotovorum*, and *Pseudomonas syringae* (Benedetti et al., 2015). OGs bind the extracellular domain of the Arabidopsis wall-associated receptor kinase1 (WAK1; Brutus et al., 2010; Gramegna et al., 2016). Treatments with OGs trigger a wide range of defense responses, including calcium increase, release of ROS and nitric oxide, production of phytoalexins, glucanase, and chitinase, and deposition of callose in the cell wall (Davis et al., 1986; Davis and Hahlbrock, 1987; Broekaert and Pneumas, 1988; Bellincampi et al., 2000; Aziz et al., 2007; Galletti et al., 2008; Rasul et al., 2012). OGs activate the transcription factors WRKY40 and WRKY33, and several genes involved in defense, such as *EDS5/SID1, SID2/ICS1, NPR1, SAG101, PAD4, ACS7, LOX3*, and *LOX4* as well as the indole glucosinolate biosynthetic genes *CYP83B1*, *CYP79B2*, *CYP79B3*, *SUR1*, and *MYB51* (Denoux et al., 2008; Souza et al., 2017). OGs activate phosphorylation of the MAP kinases AtMPK3 and AtMPK6 (Galletti et al., 2011; Mattei et al., 2016) and upregulate phenylalanine ammonia lyase, stilbene synthase (De Lorenzo et al., 1987; Aziz et al., 2007), and serine-protease inhibitors (Ferrari et al., 2003). Treatments with OGs increase the Arabidopsis resistance to *B. cinerea* through the expression of *PAD3*, which is required for the basal resistance to this fungus (Aziz et al., 2004), and, if applied to roots, induce systemic resistance in tomato plants against *B. cinerea* (Gamir et al., 2020).

### Cellodextrins

Cellodextrins are linear *β*-1,4 gluco-oligosaccharides with a degree of polymerization (DP) ranging from 3 to 9 derived from cellulose fragmentation. In grapevine, a CD with a DP of seven induces rapid ROS production and transient elevation of cytosolic calcium levels. Notably, grapevine cells pretreated with CD7 are refractory to a successive treatment with the same oligomer but respond to a subsequent application of OGs, suggesting that CDs and OGs activate different signaling pathways. CDs upregulate the expression of defense genes encoding phenylalanine ammonia lyase, stilbene synthase, chitinases, glucanases, and serine-protease inhibitors, and enhance the resistance of plants against *B. cinerea* (Aziz et al., 2007). In Arabidopsis, cellobiose (DP 2), cellotriose (DP 3), and cellotetraose (DP 4) cause an increase of intracellular calcium in a fast and transitory way, the early activation of MPK3 and MPK6, and the upregulation of some defense-related genes like those involved in the biosynthesis of glucosinolates. Cellotriose derived from the endophytic fungus *Piriformospora indica* also elicits plant defense responses (Johnson et al., 2018). Treatment with cellobiose has been reported to induce resistance to *P. syringae* pv *tomato* DC3000 (Souza et al., 2017). On the other hand, seedlings grown on high levels of cellobiose exhibit an increase in biomass and the induction of *β*-glucosidase that provides glucose as a useful carbon source for the growth of the plant itself, as well as for microbes. This is in contrast with the notion that the activation of defenses impairs growth and casts some doubts on the activity of cellobiose as a DAMP (Souza et al., 2017). Indeed, in a study, on the activity of a berberine bridge enzyme-like (BBE-like) oxidase (see below), only CDs with DP higher than two showed a significant elicitor activity (Locci et al., 2019).

### Xyloglucans

Xyloglucan oligomers have been recently identified as DAMPs in both grapevine and Arabidopsis. In Arabidopsis, they induce phosphorylation of MPK3 and MPK6, PMR4-dependent callose deposition, enhanced expression of defense genes, including *PR1*, *PAD3*, *PR2*, and *PLANT DEFENSIN 1.2* (*PDF1.2*) as well as an increased jasmonic acid (JA)-, salicylic acid (SA)-, and ethylene (ET)-dependent resistance against *B. cinerea*. In grapevine, XG oligomers induce accumulation of the phytoalexin resveratrol and resistance against *B. cinerea* (Claverie et al., 2018; Heloir et al., 2019).

### Arabinoxylans

Arabinoxylan oligosaccharides have been recently identified as novel DAMPs (Mélida et al., 2020). This group of hemicelluloses consists of a main backbone of *β*-1,4-linked D-xylose residues decorated with single L-arabinose residues linked to the C2/C3 position of a D-xylose unit (Ebringerová and Heinze, 2000). The pentasaccharide XA3XX triggers an oxidative burst, a rapid calcium influx, the phosphorylation of MPK3 and MPK6, and the upregulation of genes involved in innate immunity, including several PTI marker genes (*CYP81F2*, *WRKY53*, *PHI1*, *FRK1*, and *NHL10*). Moreover, tomato plants treated with XA3XX are more resistant to *P. syringae* pv *tomato* DC3000, and XA3XX-treated pepper plants are more resistant to *Sclerotinia sclerotiorum* (Mélida et al., 2020).

### β-1,3-Glucans

Arabidopsis plants treated with 1,3-β-d-(Glc)_6_ isolated from the pathogenic fungus *Plectosphaerella cucumerina* show the CERK1-mediated expression of several immunity-associated responses like elevation of cytoplasmic calcium concentration and MAPK cascades (Mélida et al., 2018). Because β-1,3-glucans are present in the callose of the plant papillae that are formed at the sites of infection, 1,3-β-d-(Glc)_6_ may be considered as both MAMP and DAMP.

### Oligomannans

Oligomannans with DP 2–6 enhance defense responses and expression of genes related with NO and ROS accumulation as well as the expression of *PR-1* and *LOX* in both *Nicotiana benthamiana* and rice. In rice, MOS trigger the upregulation of the expression of *MAPK12* and *MAPK6*, and lead to the accumulation of phytoalexins. Treatments with MOS protect rice and tobacco against *Xanthomonas oryzae* and *Phytophthora nicotianae*, respectively (Zang et al., 2019).

## Pathways and Signals Involved in the Growth-Defense Trade-Off

Due to their energy cost and consequent metabolic limitations, growth and defense are constantly regulated by a balanced trade-off of antithetic pathways, both of which are most likely affected by stimulatory and inhibitory signals (Huot et al., 2014; Vaahtera et al., 2019). As stated above, homeostatic control of the defense response is important to avoid deleterious effects due to a hyper-immune response that causes reduced growth and/or extensive cellular death. Under such circumstances, attenuation of the pathways leading to defense or a reduction in the levels of the signals triggering such pathways is expected to occur (Karasov et al., 2017; Li et al., 2020). The crosstalk between the pathways controlled by ET, JA, SA, and by other growth regulators may influence immune and developmental processes in opposite directions (Denancé et al., 2013; Guo et al., 2018). Over-expression of pathways that activate immunity often enhances pathogen resistance while negatively affecting plant growth and may cause anatomical and physiological responses, such as dwarfism, accelerated senescence, delayed flowering, sterility, or reduced seed production.

Many other signals, besides ET, JA, and SA, are known to influence the growth/defense trade-off. ROS in the apoplast are among these signals as their concentration and homeostasis is critical for maintaining the correct balance between growth and defense (Farvardin et al., 2020). A low level of H_2_O_2_ in the cell wall, for example, promotes growth and concomitantly suppresses the plant defense (Neuser et al., 2019). Other signals such as OGs antagonize auxin by inhibiting adventitious root formation, stem elongation, and pericycle cell differentiation. At the molecular level, OGs downregulate auxin-induced expression of the *DR5* promoter as well as the early auxin-regulated genes *IAA5*, *IAA19*, *IAA20*, *IAA22*, *SAUR16*, *SAUR AC1*, and *GH3.3*. On the other hand, auxin antagonizes the protection exerted by OGs against *B. cinerea* (Branca et al., 1988; Bellincampi et al., 1993; Ferrari et al., 2008; Savatin et al., 2011). Furthermore, a high level of endogenous OGs strongly reduces growth while a systemic and prolonged accumulation of OGs causes a hypersensitive-like response characterized by extensive cell death (Benedetti et al., 2015). Interestingly, OGs-auxin antagonism is independent of the extracellular accumulation of H_2_O_2_ produced by NADPH oxidase (Bellincampi et al., 1996, 2000) and is also independent of ET, JA, and SA signaling. The antagonism takes place downstream in the auxin signaling pathway, most likely at the post-translational level (Savatin et al., 2011).

## Oligogalacturonides and Cellodextrins are Enzymatically Oxidized by BBE-Like Proteins

**Figure 3:**
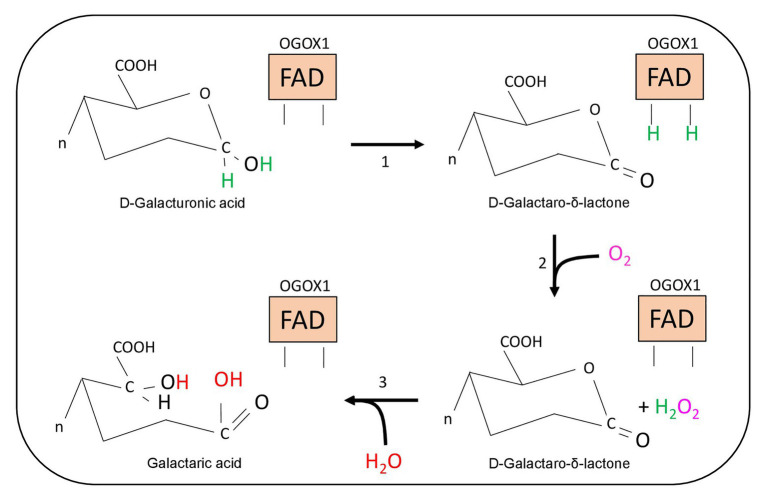
Schematic representation of the enzymatic reaction catalyzed by OGOX1. The oxidized FAD-cofactor of OGOX1 oxidizes the galacturonic acid to a lactone at the reducing-end of OGs (step 1). Molecular oxygen oxidizes the reduced FAD(H_2_)-cofactor and releases hydrogen peroxide (step 2). The final hydrolysis transforms the lactone into galactaric acid (step 3).

**Figure 4:**
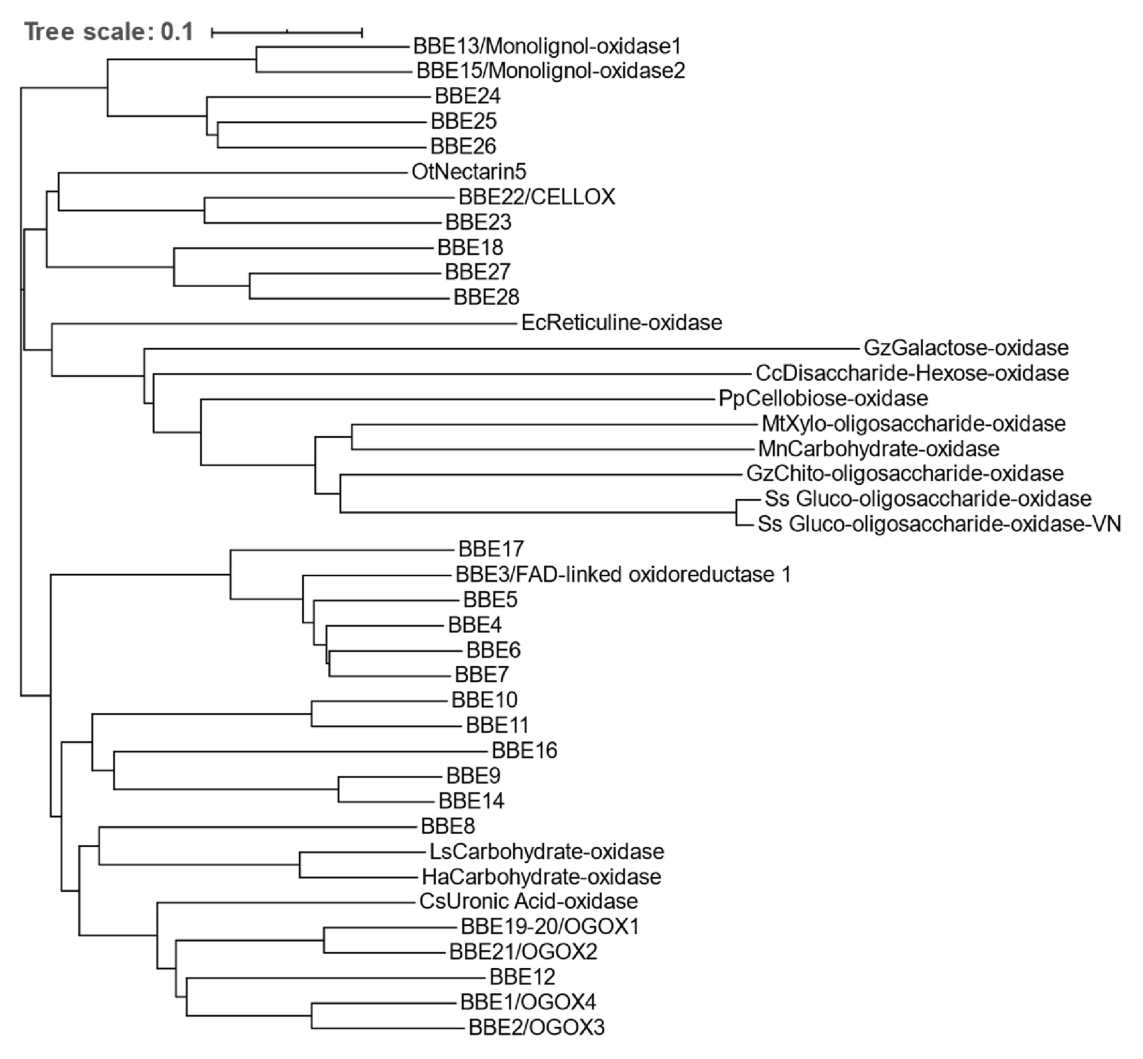
Homology tree of BBE-like proteins and FAD-linked oxidases. The BBE-like superfamily of *A. thaliana* (BBE1-28) and FAD-linked oxidases with mono- and oligo-saccharide oxidase activity from fungi, moss, and plants are indicated in the protein homology tree. Reticuline oxidase from *Eschscholzia californica* is also indicated. The identification code of each flavoprotein is reported in Table 1. (Cc, *Chondrus crispus*; Cs, *Citrus sinensis*; Ec, *Eschscholzia californica*; Gz, *Gibberella zeae*; Ha, *Helianthus annuus*; Ls, *Lactuca sativa*; Mn, *Microdochium nivale*; Mt, *Myceliophthora thermophila*; Ot, Ornamental tobacco – *Nicotiana langsdorffii x N. sanderae*; Pp, *Physcomitrella patens*; Ss, *Sarocladium strictum*; CELLOX, cellodextrin oxidase; and OGOX, oligogalacturonide oxidase).

Oxidized OGs were first identified in leaf diffusates of transgenic plants expressing the “OG-machine,” a chimeric protein generated by fusing a fungal endo-polygalacturonase with a plant PGIP (Benedetti et al., 2018). The OG-machine is capable of releasing elicitor-active OGs on command under the control of a chemically inducible promoter (Benedetti et al., 2015). The analysis of these transgenic plants revealed the presence of atypical oligomers displaying oxygen at the C1 position of the reducing end due to the conversion of a galacturonic acid into galactaric acid (Figure 3; Benedetti et al., 2018). A flavin adenine dinucleotide (FAD)-dependent and sulfite-sensitive enzyme, named oligogalacturonide oxidase (OGOX)1, capable of oxidizing OGs, was subsequently purified and found to belong to the family of BBE-like proteins, which consists of 28 members in Arabidopsis considering that one of the 27 genes, i.e., *At4g20830*, encodes for two OGOX1 isoforms, BBE19 and BBE20 (Benedetti et al., 2018; Table 1; Figure 4). OGOX1 specifically oxidizes OGs releasing H_2_O_2_ derived from the oxidization of the reduced FAD cofactor by O_2_ that, in turn, restores the activity of the enzyme (Figure 3). Three additional BBE-like proteins of the Arabidopsis BBE family, OGOX2, OGOX3, and OGOX4, oxidize OGs at a different pH (Benedetti et al., 2018). Another member of the gene family encodes an enzyme that catalyzes the conversion of indole cyanohydrin to indole-3-carbonyl nitrile, a metabolite with a role in defense (Boudsocq et al., 2010; Rajniak et al., 2015), whereas two other BBE proteins encode monolignol oxidases (Daniel et al., 2015). Finally, a BBE protein member named Cellodextrin Oxidase (CELLOX) was shown to oxidize and convert the glucose at the reducing end of CDs into gluconic acid (Locci et al., 2019). Another BBE protein, BBE23, is characterized by a 62.5% amino acid identity with CELLOX and is likely a potential paralog. CELLOX and BBE23 display the highest amino acid identity with Nectarin V, a glucose oxidase from ornamental tobacco (*Nicotiana langsdorffii x Nicotiana sanderae*; Carter and Thornburg, 2004; Table 1; Figure 4). Recently, BBE8 was hypothesized to oxidize some wall-derived oligosaccharides in guard cells in order to facilitate stomatal opening in response to infection by *P. syringae* and *Salmonella enterica* (Rodrigues Oblessuc et al., 2019). However, the activity of BBE8 as an oxidase of oligosaccharides was not proven. Similarly, the substrate specificity of BBE28 has not been identified yet despite the fact that the crystal structure of the enzyme has been solved (Daniel et al., 2016). So far, the substrates of 19 out of the 28 Arabidopsis BBE-like family members have not been identified (Table 1). The scenario of undetermined functions of these BBE-like enzymes becomes even more complex if plants with larger BBE-like families are considered such as, for example, the Western Poplar (*Populus trichocarpa*), which comprises 64 BBE-like members (Wallner et al., 2012).

**Table 1:** Berberine bridge enzyme-like (BBE-like) proteins and FAD-linked oxidases.

Identification code	Gene/protein	Substrate	Organism	Reference
At1g01980	BBE1/OGOX4	Oligogalacturonides	*Arabidopsis thaliana*	Benedetti et al., 2018
At1g11770	BBE2/OGOX3			
At1g26380	BBE3/Fad-linked Oxidoreductase 1	Indole-cyanohydrin		Rajniak et al., 2015
At1g26390	BBE4	nd		na
At1g26400	BBE5			
At1g26410	BBE6			
At1g26420	BBE7			
At1g30700	BBE8			Rodrigues Oblessuc et al., 2019
At1g30710	BBE9			na
At1g30720	BBE10			
At1g30730	BBE11			
At1g30740	BBE12			
At1g30760	BBE13/Monolignol-oxidase 1	Coumaryl alcohol Sinapyl alcohol Coniferyl alcohol		Daniel et al., 2015
At1g34575	BBE14	nd		na
At2g34790	BBE15/Monolignol-oxidase 2	Coumaryl alcohol Sinapyl alcohol Coniferyl alcohol		Daniel et al., 2015
At2g34810	BBE16	nd		na
At4g20800	BBE17			
At4g20820	BBE18			
At4g20830	BBE19-BBE20/OGOX1	Oligogalacturonides Tri-galacturonic acid Di-galacturonic acid		Benedetti et al., 2018
At4g20840	BBE21/OGOX2	Oligogalacturonides		
At4g20860	BBE22/CELLOX	Cellohexaose Cellopentaose Cellotetraose Cellotriose Cellobiose		Locci et al., 2019
At5g44360	BBE23	nd		na
At5g44380	BBE24			
At5g44390	BBE25			
At5g44400	BBE26			
At5g44410	BBE27			
At5g44440	BBE28			Daniel et al., 2016
A0A2K1JP57	BBE1/Cellobiose-oxidase	Lactose Cellobiose	*Physcomitrella patens*	Toplak et al., 2018
Q84N20 + Q84N21	NEC5/Nectarin 5	D-Glucose	*Nicotiana langsdorffii* X *Nicotiana sanderae*	Carter and Thornburg, 2004
Q8SA60	Carbohydrate oxidase	Cellopentaose Cellotetraose Cellotriose Cellobiose Lactose Maltose D-Glucose D-Galactose D-Mannose D-Atrose	*Lactuca sativa*	Custers et al., 2004
Q8SA59			*Helianthus annuus*	
P30986	BBE1/Reticuline oxidase	(S)-reticuline	*Eschscholzia californica*	Winkler et al., 2006
A0A067GII5	CISIN_1g043104mg/uronic acid oxidase	D-Galacturonic acid	*Citrus sinensis*	Wei et al., 2020
Q6PW77	Gluco-oligosaccharide oxidase	Cellopentaose Cellotetraose Cellotriose Cellobiose Maltoheptaose Maltohexaose Maltopentaose Maltotriose Maltobiose Maltose Lactose D-Glucose	*Sarocladium strictum*	Lee et al., 2005
D7PF15	Gluco-oligosaccharide oxidase-VN	Xylotriose Xylobiose Cellotriose Cellobiose Maltose D-Glucose D-Mannose N-acetyl-Glucosamine D-Galactose D-Xylose		Foumani et al., 2011
I1S2K2	Chito-oligosaccharide oxidase	Chitotetraose Chitotriose Chitobiose Cellotetraose Cellotriose Cellobiose Lactose Maltose D-Glucose N-acetyl-Glucosamine	*Gibberella zeae*	Heuts et al., 2007
P0CS93	Galactose oxidase	D-Galactose Methyl-D-Galactose Dihydroxyacetone Lactose Raffinose Melibiose Lactobionic acid		Choosri et al., 2011
G2QG48	Xylo-oligosaccharide oxidase	Xylotetraose Xylotriose Xylobiose Lactose Cellobiose D-Xylose	*Myceliophthora thermophila*	Ferrari et al., 2016
I1SB12	Carbohydrate oxidase	Maltose Cellobiose Lactose D-Glucose Deoxy-Glucose D-Galactose D-Mannose D-Xylose	*Microdochium nivale*	Kulys et al., 2001
P93762	Hexose (and Disaccharide) Oxidase	Cellobiose Maltose Lactose D-Glucose D-Galactose	*Chondrus crispus*	Groen et al., 1997

Nomenclature of BBE-like proteins from *Arabidopsis thaliana* is in accordance with TAIR database (www.arabidopsis.org/) whereas the other FAD-linked oxidases are identified by their UNIPROT code (www.uniprot.org/). The substrate of each flavoenzyme is indicated. The amino acid sequences were used to generate the protein homology tree shown in Figure 4. (CELLOX, Cellodextrin Oxidase; nd, not determined; na, not available; OGOX, Oligogalacturonide Oxidase).

## Oxidases of Mono- and Oligosaccharides in Plants and Microbes

Oxidases of mono- and oligosaccharides are widespread among plants and microbes but only in a few cases their physiological role has been investigated. To date, OGs, CDs, XG, and AX fragments, 1,3-*β*-d-(Glc)_6_ and MOS are the oligosaccharides shown to display a DAMP activity (see above). However, FAD-dependent oxidases have only been found for two of these oligosaccharides (OGs and CDs). On the other hand, (oligo)saccharides that apparently do not have DAMP activity have been found to be substrates of enzymatic oxidation both in plants and in microbes, such as *Gibberella zeae*, *Sarocladium strictum*, and *Myceliophthora thermophila*, as well as in the moss *Physcomitrella patens* (Table 1; Figure 4; Toplak et al., 2018). *Sarocladium strictum*, for example, produces mono- and disaccharide oxidases as well as gluco- and xylo-oligosaccharide oxidases which, despite sharing a high sequence identity with each other, are characterized by different substrate specificities (Lee et al., 2005; Vuong et al., 2013; Table 1). An enzyme from *G. zeae* (Figure 4; Heuts et al., 2007) oxidizes chito-oligosaccharides derived from fungal cell walls that are known to be powerful elicitors of plant defense (Wan et al., 2008). However, it is not known whether the chito-oligomers lose their elicitor activity upon oxidation as in the case OGs and CDs. Finally, an oxidase from *M. thermophila* exhibits a strong substrate preference toward xylo-oligosaccharides (Ferrari et al., 2016).

A potential physiological role of microbial FAD-dependent oligosaccharide oxidases is to provide H_2_O_2_ to support the hydrolysis of substrates, such as crystalline cellulose (Villares et al., 2017), chitin, and xylan by enzymes referred to as lytic polysaccharide mono-oxygenases (LPMOs; Nakagawa et al., 2015; Couturier et al., 2018). The copper-containing active site of LPMOs must be reduced after each oxidative-cleavage reaction and electrons may be restored by the H_2_O_2_ produced by the FAD-depended enzymes (Filandr et al., 2020; Giovannoni et al., 2020).

In conclusion, oxidases of mono- and oligosaccharides are widely distributed among plants and microbes, but their role is difficult to decipher and may depend on the nature of the producing organism, the substrate specificity of the enzyme, and the metabolic role of the substrate. The plant-encoded oxidizing enzymes acting on DAMPs can attenuate the excessive activity of these elicitors of defense. The same kind of enzymes produced by pathogens may sustain the activity of microbial LPMOs in the degradation of resistant cell wall substrates such as crystalline cellulose. Pathogenic microorganisms may recruit the plant derived FAD-dependent oxidases to sustain the activity of LPMOs and cause a diversion (hijacking) of the host responses into an advantageous trait for the pathogens.

## Final Considerations: The Role of Oxidases in the Maintenance of Signal Homeostasis and Growth-Defense Trade-Off

As a co-product of the enzymatic oxidation of OGs, CDs, and possibly of other DAMPs, H_2_O_2_ is expected to be present temporally and locally in limited zones of the tissues, where a breach in the wall is generated. This may occur upon local injury caused by biotic or abiotic stress or upon a localized loss of CWI during growth and development like, for example, during the formation of lateral roots (Peretto et al., 1992). It is known that H_2_O_2_ is involved not only in the strengthening and repairing of the plant tissues (Farvardin et al., 2020) but also in signaling during both immunity and development (Mhamdi and Van Breusegem, 2018; Huang et al., 2019; Sies and Jones, 2020). Spatial distribution of different ROS and their finely-tuned balance drive plant morphogenetic processes (Mhamdi and Van Breusegem, 2018). It can be argued that the enzymatic oxidation of wall polysaccharide fragments by the FAD-dependent oxidases is also a mechanism for a strictly localized production of limited amounts of H_2_O_2_. This production of H_2_O_2_ is expected to occur only where breaks are made by CWDEs in the wall, and therefore only in one or few cells, and may have a biological significance, for example, as a signal for a very short range cell-cell communication. In addition, the oxidation of the reducing end of the cell wall polysaccharides may hamper the trans-glycosylation that is critical for cell expansion (Franková and Fry, 2020), thereby contributing to the growth-defense trade-off.

Both OGs and CDs play a dual function as elicitors of plant immunity and as a carbon source sustaining the growth of phytopathogenic microbes. Oxidation of these oligosaccharides protects against hyper-immunity and blocks pathogen growth by making them more difficult to metabolize. Consequently, transgenic Arabidopsis plants overexpressing OGOX1 and CELLOX are more resistant to *B. cinerea* because the fungus does not grow well when fed with a mixture of oxidized oligosaccharides (Benedetti et al., 2018; Locci et al., 2019). It is relevant, in this context, that a marked accumulation of oxidized di-galacturonic acid has been detected as a final product of tissue degradation in Arabidopsis plants infected with *B. cinerea* (Voxeur et al., 2019). The recalcitrance to enzymatic hydrolysis of the oxidized oligosaccharides is also favored by the basification of the apoplastic pH normally occurring as a defense response to microbial attacks. OGOXs and CELLOX display a high optimum pH of activity while the microbial pectinases and cellulases display an optimal activity between pH 3 and 6.

The recognition of DAMPs by pattern recognition receptors is thought to occur ubiquitously across the tree of life (Heil and Land, 2014; De Lorenzo et al., 2018). In mammals and plants, DAMP-mediated immunity exhibits both common and divergent features (De Lorenzo et al., 2018). Elicitors such as DAMPs and MAMPs elicit what is referred to as PTI. Attenuation or suppression of PTI is expected to protect the organism from the deleterious effects of hyperactivated immunity that negatively affect growth (Li et al., 2020). Currently, attenuation of PTI, through phosphorylation and dephosphorylation of the MAPKs, protein degradation by the proteasome, or regulation of WRKY transcription factors is known to occur downstream of MAMP perception (Li et al., 2020). However, not much is known about the attenuation/clearance of DAMPs that must occur to avoid a continuous activation of defense once a corresponding danger has ceased to exist. Moreover, the homeostasis of endogenous molecules that normally have a physiological role and become DAMPs upon pathogen attack needs to be quickly restored, as in the case of OGs that are released in plants not only upon microbial attacks but also in the healthy plants possibly to regulate growth and development in concert with hormones (Pontiggia et al., 2015).

**Figure 5:**
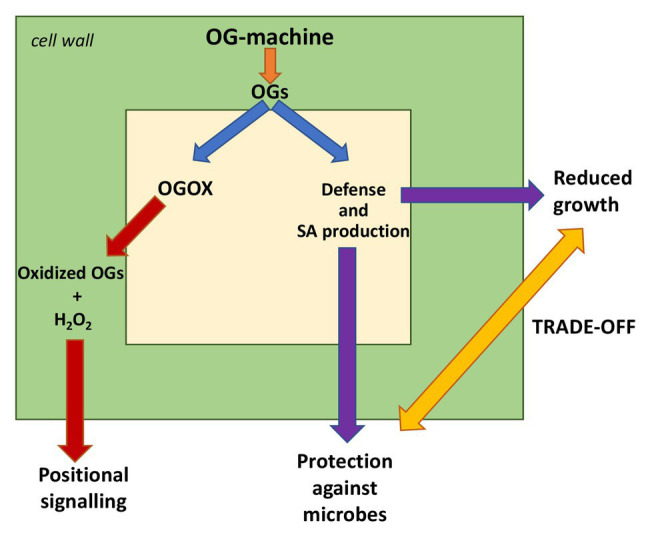
Cellular responses to the “OG-machine” action. The plants expressing the OG-machine under an inducible promoter produce OGs on command. These activate defenses and salicylic acid (SA) formation that, on one side, confer protection against pathogenic fungi and bacteria and, on the other side, reduce the plant growth (trade-off). Excess of accumulated OGs causes cell death (hyper-immunity). Homeostasis of OGs is maintained by an oxidase (OGOX) producing hydrogen peroxide that in turn may act as a transduction signal. Oxidized OGs cannot be utilized by some microbes as a carbon source; this contributes to plant resistance.

The complexity of the cell wall does not allow an easy *in vivo* investigation of the release, activity, or degradation of oligosaccharide fragments. To this purpose, an excellent tool by which the cause-effect of the biological activity of OGs has been clearly documented is the “OG-machine” expressed in Arabidopsis. Using these plants, it is possible to release elicitor-active OGs under the control of an inducible promoter. Released OGs *in vivo* activate immunity, confer protection against fungal and bacterial pathogens, and, if accumulated in excess, cause an increase of salicylic acid, reduction of growth, leaf discoloration, and chlorosis and, finally, cell death; i.e., a typical hypersensitive response (Figure 5; Benedetti et al., 2015). The first oxidase specifically acting on DAMPs was identified in these plants, where the enzyme is constitutively expressed (Benedetti et al., 2018). The same plants not only express high levels of OG-oxidases but also an oxidase acting on CDs (Locci et al., 2019). Notably, recent studies show that cellulose and pectin interact (Du et al., 2020; Palacio-Lopez et al., 2020) and that cellulose microfibers and HG nanofilaments form a single cohesive network (Haas et al., 2020; Zhang and Zhang, 2020).

Oxidases can attenuate DAMP activity of OGs and CDs but a relevant question is why plants have evolved such a type of activity if they can potentially mitigate or abolish the DAMP signal by using the vast variety of endogenous degrading enzymes (pectinases and cellulases). One possible answer is found in the evidence that the final products of hydrolysis of pectin and cellulose are utilized as a carbon source by microbes, and therefore may contribute to plant susceptibility. The combination of hydrolases and oxidases, instead, produces oligosaccharide fragments that cannot be readily catabolized by microbes and, consequently, contributes to enhance the plant resistance (Locci et al., 2019).

In humans, inflammation is not only caused by a hyper-accumulation of inducible DAMPs but also by an imbalance of suppression/inhibition of DAMPs or by an insufficient generation of the so-called “SAMPs,” where “S” stands for “suppressing DAMPs” (Land, 2018; Relja and Land, 2020). SAMPs include molecules such as prostaglandin E2 (PGE2), annexin A1 (AnxA1), and specialized pro-resolving mediators (SPMs; Land, 2018). The attention on SAMPs is increasing because of their potential therapeutic applications in pathological processes of hyper-inflammation like those currently observed in patients affected by COVID-19, and which, in certain cases, may lead to severe autoimmune disorders (Gallo et al., 2015; Land, 2020). DAMP oxidases can be considered in the broadest sense as DAMP suppressors: i.e. SAMPs that maintain a balanced level of signals in plants. The identification of plant SAMPs may have significant biotechnological applications to overcome the limitations imposed by the growth/defense trade-off and by an excessive accumulation of DAMPs causing a deleterious hyper-immunity.

## Author Contributions

DP, MB, and SC contributed to writing. FC and GL supervised the work and edited the final version of the paper. All authors contributed to the article and approved the submitted version.

### Conflict of Interest

The authors declare that the research was conducted in the absence of any commercial or financial relationships that could be construed as a potential conflict of interest.
